# All that Glitters is not Cholecystitis. A Rare Presentation of Acute Pericarditis Mimicking Cholecystitis and Review of the Literature

**DOI:** 10.15388/Amed.2022.29.2.8

**Published:** 2022-06-29

**Authors:** Anna Garmpi, Christos Damaskos, Nikolaos Garmpis, Vasiliki E. Georgakopoulou, Vaios Vasileios Kaminiotis, Evangelos Diamantis, Alexandros Patsouras, Athanasios Syllaios, Dimitrios Dimitroulis

**Affiliations:** First Department of Propedeutic Internal Medicine, Laiko General Hospital, Medical School, National and Kapodistrian University of Athens, Athens, Greece; Renal Transplantation Unit, Laiko General Hospital, Athens, Greece; N.S. Christeas Laboratory of Experimental Surgery and Surgical Research, Medical School, National and Kapodistrian University of Athens, Athens, Greece; N.S. Christeas Laboratory of Experimental Surgery and Surgical Research, Medical School, National and Kapodistrian University of Athens, Athens, Greece; Second Department of Propedeutic Surgery, Laiko General Hospital, Medical School, National and Kapodistrian University of Athens, Athens, Greece; Department of Pulmonology, Laiko General Hospital, Athens, Greece; Cardiothoracic Department, Derriford Hospital, University Hospitals Plymouth, Plymouth, United Kingdom;; Academic Department of Internal Medicine - Endocrinology Unit, Agioi Anargyroi General Oncology Hospital of Kifisia, National and Kapodistrian University of Athens Athens Greece; Second Department of Pulmonology, Sotiria Hospital, Athens, Greece; First Department of Surgery, Laiko General Hospital, Medical School, National and Kapodistrian University of Athens, Athens, Greece; Second Department of Propedeutic Surgery, Laiko General Hospital, Medical School, National and Kapodistrian University of Athens, Athens, Greece

**Keywords:** pericarditis, pericardial effusion, heart failure, gallbladder edema, acalculous cholecystitis

## Abstract

Acute pericarditis is the most common inflammatory disorder of the pericardium, responsible for approximately 5% of visits to the emergency departments, concerning chest pain without myocardial infarction. We report a case of a 41-year-old man who presented to our hospital, complaining about retrosternal and epigastrium pain. The transthoracic echocardiogram showed pericardial effusion while the electrocardiogram and laboratory findings revealed acute pericarditis. An abdominal ultrasound revealed gallbladder edema. The pericardial effusion was treated with pericardial catheter insertion, diuretics, and nonsteroidal anti-inflammatory drugs. This case shows that acute pericarditis can be clinically presented with many ways, one of them being gallbladder edema. Furthermore, in this case-based review we present all cases of simultaneous appearance of pericarditis and acalculous cholecystitis or gallbladder edema.

## Santrauka.

Ūminis perikarditas yra dažniausias uždegiminis širdiplėvės sutrikimas, nustatomas maždaug 5 proc. atvejų, pacientams patekus į skubios pagalbos skyrių, kai jaučiamas krūtinės skausmas, tačiau nenustatoma miokardo infarkto. Pateikiame 41 metų vyro, atvežto į mūsų ligoninę, atvejį. Jis skundėsi retrosterniniu ir epigastriniu skausmu. Perstemplinės echokardiografijos tyrimas atskleidė perikardo efuziją, o elektrokardiograma ir laboratoriniai tyrimai rodė esant ūminį perikarditą. Abdominaliniu ultragarso tyrimu nustatyta tulžies pūslės edema. Perikardo efuzija buvo gydoma į perikardo ertmę įkišus kateterį, diuretikais ir nesteroidiniais vaistais nuo uždegimo. Šis atvejis atskleidžia, kad ūminis perikarditas gali kliniškai reikštis įvairiais būdais, vienas iš kurių yra tulžies pūslės edema. Be to, kartu su šia konkretaus atvejo analize pristatome visus atvejus, kai vienu metu pasireiškė ir perikarditas, ir cholecistitas arba tulžies pūslės edema.

## Introduction

Acute pericarditis is considered the most common inflammatory disorder of the pericardium, although its true prevalence remains unknown [1, 2]. It is estimated that 5% of visits to the emergency departments concerning chest pain without myocardial infarction are due to pericarditis [3]. The median age of pericarditis development is 51 years old, with a ratio of men to women of 2:1. Female gender is related to a worse prognosis [4].

As far as the clinical presentation is concerned, the patients complain of precordial or retrosternal chest pain, which may radiate to various sites, including the neck, jaw, and shoulder. Additionally, symptoms such as cough and shortness of breath may appear. The most diagnostic clinical sign is the pericardial friction rub [5]. However, this sign is not always present during clinical examination. More than half of the patients present with a pericardial effusion on an echocardiogram [2]. The most important complication of acute pericarditis is the cardiac tamponade, which can be diagnosed by pulsus paradoxus, hypotension, and jugular distention [6]. The diffuse elevation of the ST segment is the typical electrocardiographic sign, although its existence reveals injury to the myocardium since the pericardium is avascular [7].

The imaging methods used for the detection of pericarditis initially include chest X-ray, which can be either normal or reveal an increased cardiothoracic ratio [1]. Transthoracic echocardiogram (TTE) is regarded as the most important imaging technique, which can detect both fluid and abnormal cardiac function. The use of computed tomography (CT) or magnetic resonance imaging (MRI) is restricted to specific causes of pericarditis, including tuberculosis and malignancies, when myocarditis is suspected [7]. Nevertheless, both CT and MRI can be vital in doubtful cases, as any abnormal pericardial thickening and contrast enhancement are highly suggestive of acute pericarditis.

Blood markers, such as erythrocyte segmentation rate (ESR), white blood cell (WBC) count and C-reactive protein (CRP), are usually elevated [1, 2]. The liver function tests can also be affected [7]. The troponin level may be slightly elevated, whereas its marked elevation reveals myocarditis [1, 2, 7]. In addition, serological tests for viruses and autoantibody screening, including anti-nuclear antibodies (ANA) and rheumatoid factor (RF), may be conducted since viruses and rheumatological disorders can cause this disease [7].

As aforementioned, the causes of pericarditis are various and can be divided into idiopathic, infectious, inflammatory, and autoimmune [8-11]. The treatment and management of this disease is defined by its underlying etiology [7].

Herein, we report a rare case of acute pericarditis mimicking acalculous cholecystitis as it was presented with signs and symptoms of gallbladder edema. In addition, we present all the cases reported in the literature of pericarditis presenting as gallbladder edema or mimicking acalculous cholecystitis.

## Case presentation

A 41-year-old male patient was referred to our emergency department with complaints of malaise, cough, retrosternal chest pain and epigastrium pain lasting one month. For this reason, he had undergone a gastroscopy one month earlier, without any abnormal findings, and an abdominal ultrasound (US) which, at that time, had revealed a gallbladder edema. The US could not be interpreted adequately though, as it was performed in a nonfasting state and the gallbladder was contracted. He also reported an upper respiratory infection treated with nonsteroidal anti-inflammatory drugs (NSAID) 6 weeks ago. His had no significant past medical history, and no substance abuse or travelling abroad was mentioned.

On admission, his blood pressure was within the normal range (135/82 mmHg), he had a heart rate of 95 bpm, respiratory frequency of 17 breaths/min and temperature of 37.2°C. His oxygen saturation was 98% on room air. The physical examination revealed mild epigastrium tenderness without the presence of Murphy’s sign nor palpable liver. In addition, the respiratory sounds were slightly diminished on the left lung. As far as the heart sounds are concerned, no abnormalities were noticed and no signs of heart failure, such as distention of jugular vein or peripheral edema, were observed.

A complete blood count demonstrated WBC 11700/μL (normal 4000–11000/μL) with 68.7% neutrophils. The biochemistry tests showed a CRP of 8.6 mg/dL (normal <0.5mg/dL), aspartate transaminase (AST) 99 IU/L (normal 14–20 IU/L), alanine transaminase (ALT) 152 IU/L (normal 10–40 IU/L), gamma-glutamyl transpeptidase (GGT) 275 IU/L (normal 0–70 IU/L) and total bilirubin 0.76 mg/dL (normal 0.2–0.8mg/dL). Two blood cultures were obtained which proved sterile.

**Figure 1.**
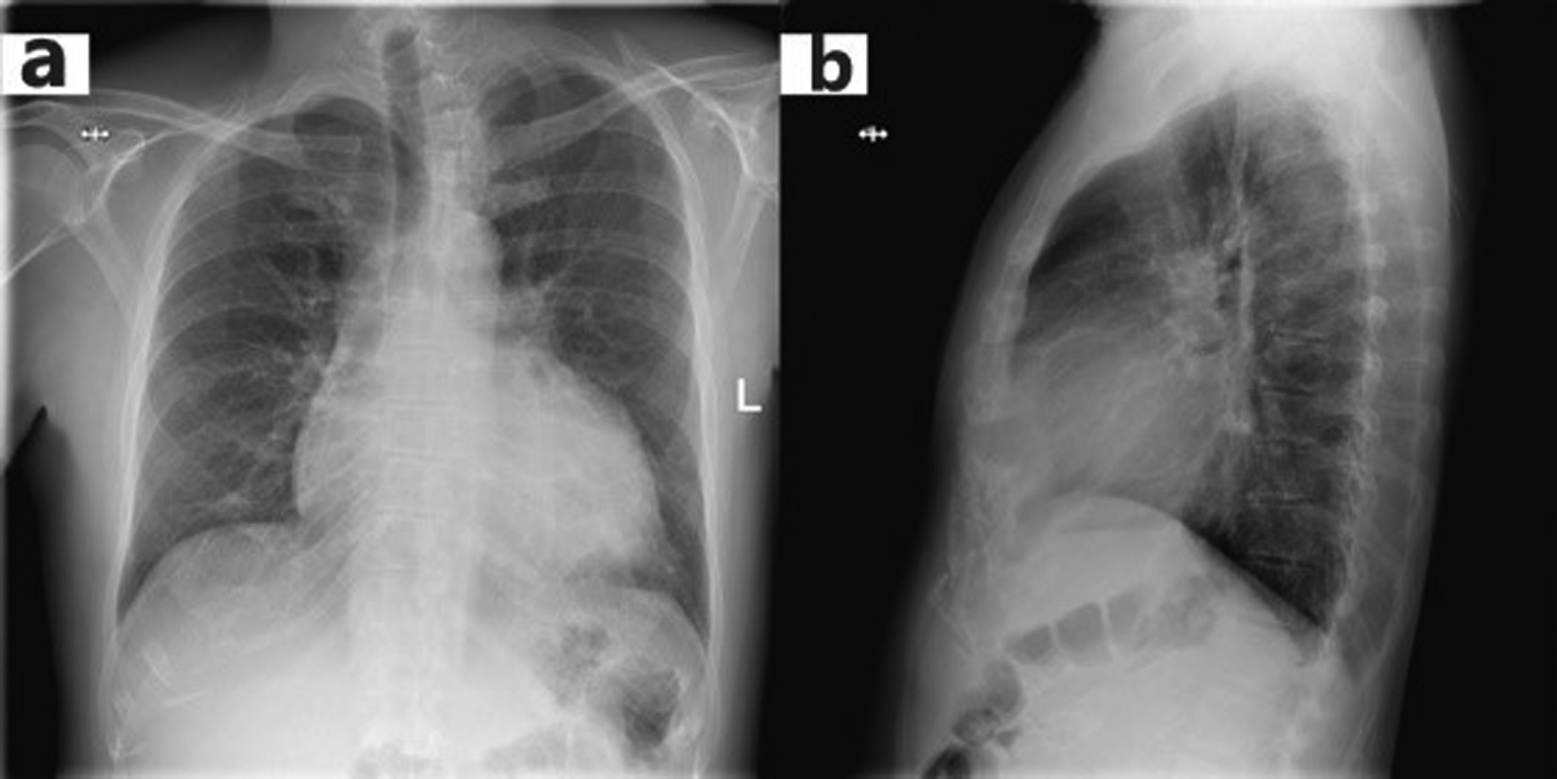
The chest X-Ray revealing cardiomegaly and increased cardiothoracic ratio (CTR). **a**: Face; **b**: Profile.

**Figure 2.**
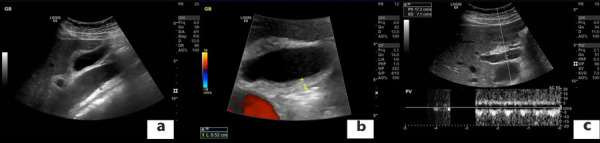
Abdominal ultrasound (US) findings. **a**, **b**: Gallbladder edema without gallstones, extra- nor intra-hepatic dilations nor pericholecystic fluid and a dilated inferior vena cava; **c**: Color doppler sonography demonstrated also reduced blood flow to the inferior vena cava.

**Figure 3.**
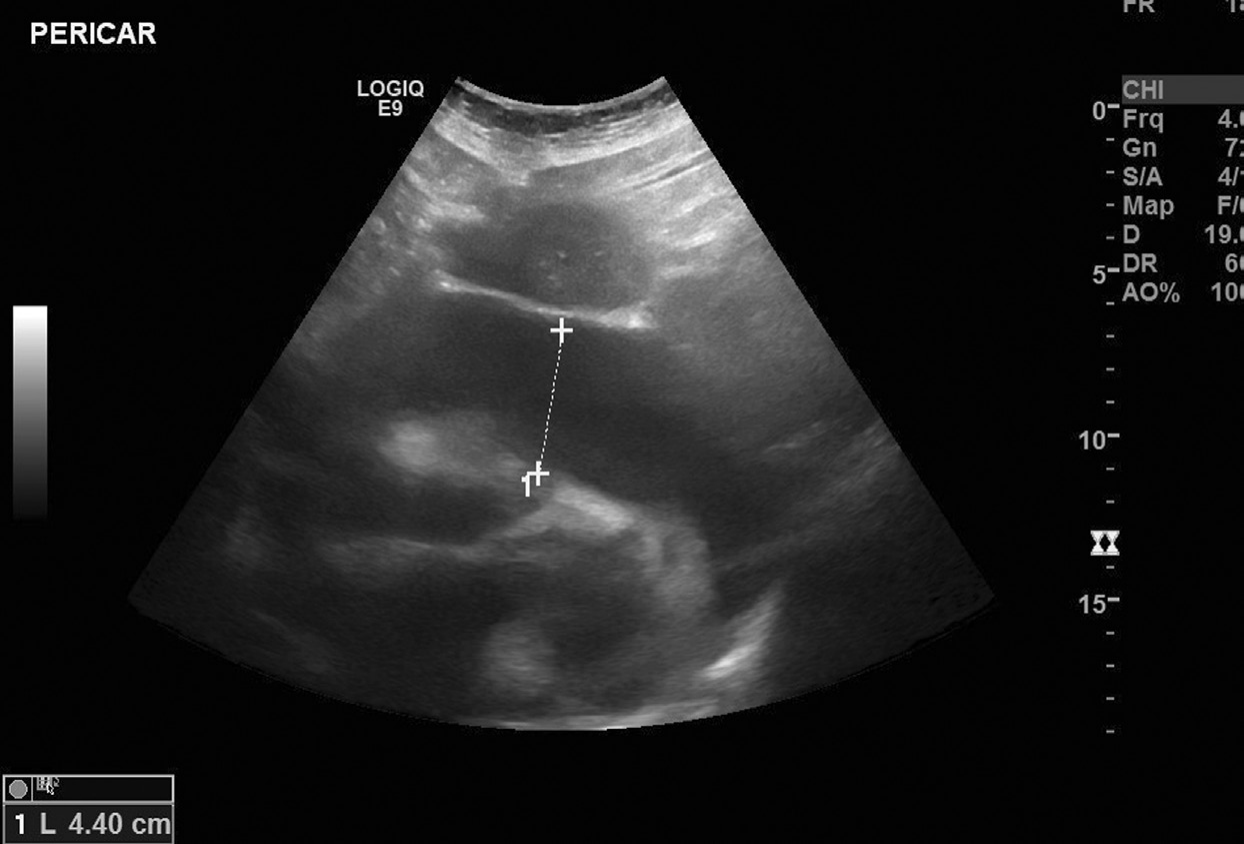
Transthoracic echocardiogram (TTE) revealing a large circumferential pericardial effusion creating an echo-free space of 4,6 cm and a swinging heart.

**Figure 4.**
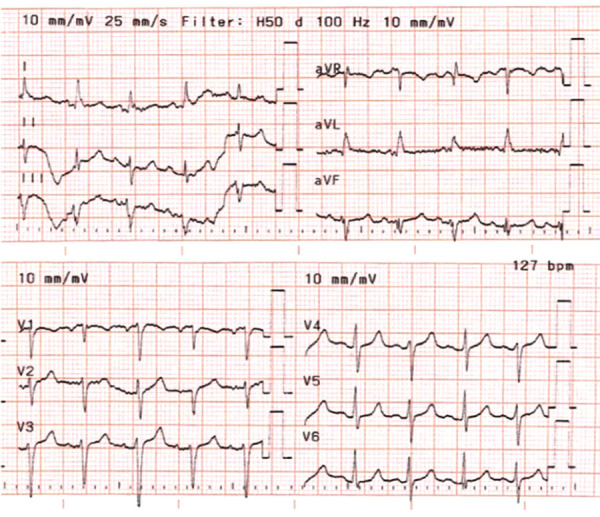
Echocardiogram (ECG) revealing sinus rhythm and diffuse ST segment elevation.

The chest X-ray revealed cardiomegaly and increased cardiothoracic ratio (CTR), Figure 1. In addition, an abdominal US was conducted, which showed gallbladder edema without gallstones, extra- nor intrahepatic dilations nor pericholecystic fluid and a dilated inferior vena cava (Figure 2a, b). Color Doppler sonography demonstrated also reduced blood flow to the inferior vena cava (Figure 2c). Therefore, a TTE was performed which revealed a large circumferential pericardial effusion creating an echo-free space of 4.6 cm and a swinging heart (Figure 3). No signs of diminished cardiac wall motion or valvular dysfunction was noticed. Only early mild right ventricular wall diastolic collapse with slightly increased respiratory variation in transvalvular flow velocities were observed while the ejection fraction was 60%. An electrocardiogram was performed afterwards, which showed sinus rhythm and diffuse ST segment elevation compatible with acute pericarditis (Figure 4). More blood tests were conducted, which revealed that the creatine phosphokinase (CPK) level was elevated (240 IU/L) (normal 22-198 IU/L) while troponin test was negative. The B-type natriuretic peptide was normal. Subsequently, these findings were consistent with acute pericarditis and the gallbladder edema was a result of venous congestion due to starting right cardiac failure.

**Figure 5.**
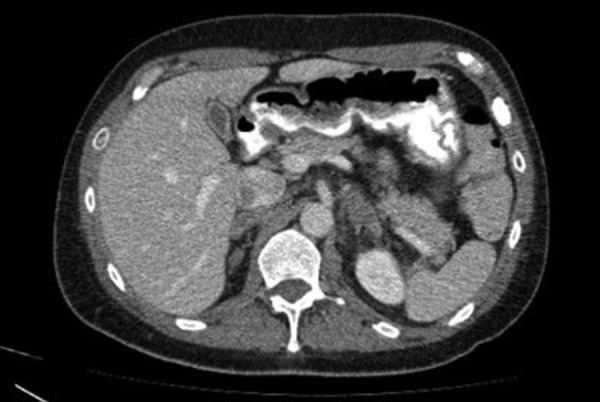
Abdominal computed tomography (CT) revealing gallbladder edema without gallstones and pericholecystic fluid.

**Figure 6.**
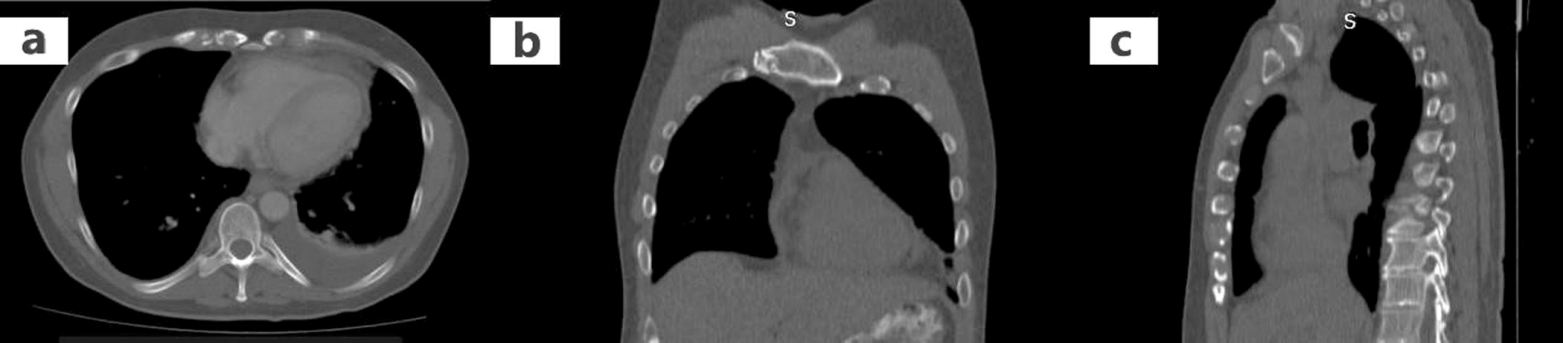
Chest computed tomography (CT) revealing a pericardial effusion and a small left pleural effusion. **a**: Transverse section; **b**: Coronal section; **c**: Sagittal section.

Further imaging evaluation with abdominal (Figure 5) and chest CT (Figure 6a-c) was performed, which showed additionally to pericardial effusion a small left pleural effusion. Finally, a pericardial catheter under ultrasound guidance was inserted, due to high risk of cardiac tamponade. Additionally, diuretics and NSAIDs were administered. The pericardial fluid was transudative and both the bacterial staining and cultures were negative. Testing for ANA and RF, and diagnostic virology tests were negative. After 20 days, both the gallbladder edema and the pericardial effusion had vanished, the liver function returned to normal, and the patient presented no symptoms.

## Discussion

This case is worth mentioning since only a few cases of pericarditis in the literature are presented with gallbladder edema that could also be misdiagnosed as acalculous cholecystitis (Table 1) [12, 13]. In 1991, Donnelly *et al*. presented two cases of purulent pericarditis in children, in which the initial manifestation was acute abdomen and the imaging findings revealed gallbladder wall thickening without cholecystitis [12]. These patients were managed with pericardiocentesis and antibiotic administration. These cases differ from our case since their cause is bacterial, including *Streptococcus pneumoniae* and *Neisseria meningitis*. In 2018, Zenda *et al*. reported a case of a 29-year-old man suffering from acute peri-myocarditis [13]. The US revealed extensive gallbladder edema combined with pericardial effusion. No specific treatment was administered to the patient [7].

It is of paramount importance to distinguish acute acalculous cholecystitis from gallbladder edema. The edema constitutes an imaging finding, which can be explained from various pathophysiological mechanisms such as congestive heart failure. On the contrary, acute acalculous cholecystitis constitutes a necrotic inflammatory process with a poor prognosis. It is associated with empyema and gangrene and other severe complications [14]. It is a rare cause of acute cholecystitis, and the clinical presentation is atypical since it mainly includes pain, fever, and leukocytosis [15]. In our case, the gallbladder edema that was a result of congestive hepatopathy due to starting right heart failure [16]. Hepatic veins blood congestion leads to hepatocellular damage due to diminished hepatic oxygenation. This explains the high levels of AST and ALT in our patient. Furthermore, the elevation of cholestatic enzymes is attributed to the bile duct compression [16, 17]. Gradual accumulation of pericardial fluid did not result in evident signs and symptoms of right heart failure.

The existence of gallbladder edema without evidence of acalculous cholecystitis is crucial for the differential diagnosis. The simultaneous existence of acalculous cholecystitis and pericardial effusion or pericarditis can reveal underlying diseases, causing these lesions (Table 2). These underlying diseases include rheumatological disorders such as lupus erythematosus or Churg–Strauss syndrome [18-20]. In addition, bacterial or parasitic infections, such as Leptospira or Ascaris, are reported in the literature [21, 22]. A case of pulmonary carcinoma, with simultaneous appearance of acalculous cholecystitis and cardiac tamponade, is also reported in the literature [23].

**Table 1.** Studies indicating the coexistence of pericarditis and gallbladder edema.

	Study	N	Sex	Age	Symptoms or signs	Method of diagnosis	Etiology	Treatment
1	Donnelly *et al.,* 1999 [12]	2	F	0,5 y	Abdominal distention, fever and signs of sepsis.	Chest X-ray, abdominal CT, pericardial fluid culture.	Streptococcus pneumoniae.	Antibiotics and pericardiocentesis.
			M	2,5 y		Chest X-ray, US, echocardiography during laparotomy, pericardial fluid culture.	Neisseria meningitis.	
2	Zenda *et al.,* 2018 [13]	1	M	29 y	Right upper quadrant pain, fever and malaise.	Chest X-Ray, electrocardiogram, blood tests and US.	Undiagnosed.	No treatment.
3	Current study	1	M	41 y	Epigastrium and retrosternal pain.	Chest X-Ray, US, echocardiography, electrocardiogram, abdominal and chest CT, blood tests, pericardial fluid culture.	Possible viral etiology.	Diuretics, non-steroid anti-inflammatory drugs and pericardiocentesis.

N: Number of patients; F: Female; y: Years; CT: Computed tomography; M: Male; US: Ultrasonography.

**Table 2.** Studies indicating the coexistence of pericarditis and acalculous cholecystitis.

	Study	Etiology		N
1	Albaladejo M *et al.,* 1995 [23]	Malignancy	Lung carcinoma	1
2	Barrera-Ramirez CF *et al.,* 2005 [20]	Rheumatological disorder	Lupus erythematosus	1
3	Kaji K *et al.,* 2007 [22]	Infection	Ascaris	1
4	Lenders G *et al.,* 2011 [18]	Rheumatological disorder	Churg-Strauss syndrome	1
5	Santos VM *et al.,* 2014 [21]	Infection	Leptospira	1
6	Obreja EL *et al.,* 2019 [19]	Rheumatological disorder	Lupus	1

N: Number of patients.

Regarding the treatment, the catheter insertion was performed since there was a significant pericardial effusion with high risk of cardiac tamponade [1, 2]. The medical therapy included NSAIDs and diuretics. The aim of therapy against acute pericarditis is the diminution of inflammatory process, symptomatic relief, and prevention of recurrence [7]. We assumed that the cause of our case is viral because the fluid was transudative, the cultures were negative, and his past medical history reports a recent upper respiratory tract infection. For that reason, we avoided the use of corticosteroids [24]. The use of colchicine is applied mostly against the appearance of recurrences [7].

## Conclusion

In conclusion, pericarditis is a common cardiological disorder which can be manifested in many ways, including acute cholecystitis and gallbladder edema. Thus, our case highlights the need for high clinical suspicion when there is a possible diagnosis of acute cholecystitis. Our case-based review is worthwhile mentioning since the simultaneous appearance of pericardial effusion and gallbladder edema can be caused by various diseases, making its investigation and appropriate treatment a challenging issue.
